# ﻿Three new species and one new record of *Scleroderma* (Sclerodermataceae, Boletales) from northern Thailand

**DOI:** 10.3897/mycokeys.123.160438

**Published:** 2025-10-08

**Authors:** Didsanutda Gonkhom, Phongeun Sysouphanthong, Marc Stadler, Naritsada Thongklang, Kevin D. Hyde

**Affiliations:** 1 School of Science, Mae Fah Luang University, Chiang Rai, Thailand; 2 Center of Excellence in Fungal Research, Mae Fah Luang University, Chiang Rai, Thailand; 3 Department of Microbial Drugs, Helmholtz Centre for Infection Research (HZI), 38124 Braunschweig, Germany; 4 German Centre for Infection Research (DZIF), partner site Hannover/Braunschweig, Inhoffenstrasse 7, 38124 Braunschweig, Germany

**Keywords:** Basidiomes, Boletales, morphology, taxonomy, three new species

## Abstract

The genus *Scleroderma* (Sclerodermataceae) contains gasteroid ectomycorrhizal fungi and is distributed worldwide in temperate and tropical regions. Fresh specimens were collected in Thailand and report three undescribed species and one new record for the country. These species were characterized by photographs of freshly collected basidiomes, and their macro- and microscopic features were compared with those of known species of *Scleroderma*. Additionally, DNA sequence data were generated for four loci, including the nuclear ribosomal internal transcribed spacer region (ITS), the large subunit ribosomal RNA gene (LSU), the translation elongation factor 1-alpha gene (*tef*1-*α*), and the second largest subunit of RNA polymerase II (*rpb*2). A multi-locus phylogeny was constructed to confirm their taxonomic placement.

## ﻿Introduction

Ectomycorrhizal fungi form symbiotic relationships with the feeder roots of many tree species and other plants that benefit both partners (Charya and Garg 2019). They are distributed worldwide in temperate and tropical regions (Corrales et al. 2018). The ectomycorrhizal fungi include the genus *Scleroderma* (Ouatiki et al. 2022). Persoon (1801) erected the genus *Scleroderma*, which was later updated by Guzmán (1970), who proposed infrageneric classifications such as sections *Sclerangium*, *Scleroderma*, and *Macrospora* based on morphology. Modern phylogenetic studies have further refined these groupings (de Menezes Filho et al. 2022; Wu et al. 2023). From early morphology-based classifications to more recent genetic investigations that have improved our understanding of species relationships (Persoon 1801; Guzmán 1970; Wu et al. 2023), the history of *Scleroderma* demonstrates the evolving dynamics of fungal taxonomy. The evolution from *Lycoperdon verrucosum* to *S. verrucosum* illustrates how scientific classification continues to evolve (Persoon 1801).

There are 206 *Scleroderma* species listed in Index Fungorum (https://www.indexfungorum.org/Names/Names.asp, accessed on 24 March 2025) and 76 species in Species Fungorum (https://www.speciesfungorum.org/Names/Names.asp, accessed on 24 March 2025). The genus belongs to the family Sclerodermataceae, order Boletales, and class Agaricomycetes (Binder and Hibbett 2006).

*Scleroderma* species have traditionally been segregated according to the morphology of the basidiomata and the surface of the peridium, the type of dehiscence of the peridium, the color of the gleba, and the ornamentation of their basidiospores (de Menezes Filho et al. 2022). Moreover, the thickness and scaliness of the peridium, the presence of stalks of the basidiome, and the form of the stipe have occasionally been used to distinguish species in the genus (Raut et al. 2020).

Although regions such as Europe and America are relatively well studied in terms of *Scleroderma* taxonomy, data are lacking for tropical Africa (Sanon et al. 1997) and Asia (Farmer and Sylvia 1998; Sims et al. 1999). In Thailand, 11 species have been reported, namely *S. areolatum*, *S. aurantium*, *S. bovista*, *S. cepa*, *S. citrinum*, *S. dictyosporum*, *S. flavidum*, *S. lycoperdoides*, *S. polyrhizum*, *S. sinnamariense*, and *S. verrucosum*, based on morphological characteristics (Chandrasrikul et al. 2011) and molecular analysis of *S. suthepense* (Kumla et al. 2013).

In our study, our aim was to describe three new species and a new record of *Scleroderma* from northern Thailand, based on macro- and microscopic characteristics and molecular phylogenetic methods.

## ﻿Materials and methods

### ﻿Sample collection

Fresh basidiomes of *Scleroderma* were collected during the rainy season from May to June 2019 in Chiang Mai and Chiang Rai provinces. A total of 21 specimens were collected, including representatives of three new species. The specimens were dried in an oven at 45 °C for at least 24 hr and stored at room temperature. They are deposited at Mae Fah Luang University Fungarium (MFLU Fungarium).

### ﻿Morphological analysis

Fresh basidiomata were described for macrocharacters, photographed in the field, and tested for macrochemical reactions (color reactions) of the peridium with 5% potassium hydroxide (KOH). Their size and color were recorded, and color was compared with the Methuen Handbook of Color (Kornerup and Wanscher 1981). For observation of microcharacters, dried samples were sectioned with razor blades, mounted on glass slides, and rehydrated with 5% KOH (w/v). The sizes and shapes of the microstructures, including hyphae, basidia, and basidiospores, were examined under a microscope (Nikon DS-Ri2). At least two specimens of each species were measured, and each characteristic was measured in at least 50 replicates. Spore ornamentation was also examined.

### ﻿DNA extraction, PCR amplification, and sequencing

Genomic DNA was extracted from 100 mg of the fruiting body using the Biospin Fungus Genomic DNA Extraction Kit (Bioer Technology, Hangzhou, China) following the manufacturer’s protocol. The polymerase chain reaction (PCR) was used to amplify the internal transcribed spacer (ITS), the 28S large subunit region of ribosomal DNA (LSU), the second largest subunit of RNA polymerase II (*rpb*2), and the translation elongation factor 1-alpha (*tef*1-*α*). The primer pairs used were: ITS1-F and ITS4 for ITS (White et al. 1990; Gardes and Bruns 1993), LR0R and LR5 for LSU (Vilgalys and Hester 1990; White et al. 1990), RPB2-6F and RPB2-7cR for *rpb*2 (Rehner and Buckley 2005), and EF1-983F and EF1-1567R for *tef*1-*α* (Rehner and Buckley 2005). The PCR cycling conditions for ITS, LSU, *rpb*2, and *tef*1-*α* were: 3 min at 94 °C; 35 cycles of 30 s at 94 °C, 30 s at 52 °C, and 1 min at 72 °C; followed by 10 min at 72 °C. For *tef*1-*α*, the program was: 5 min at 95 °C; 35 cycles of 1 min at 94 °C, 2 min at 52 °C, and 1.5 min at 72 °C; followed by 10 min at 72 °C. Sequencing of PCR-amplified products in both directions was performed by Sangon Biological Engineering Technology and Services (Shanghai).

### ﻿Alignment and phylogenetic analysis

Phylogenetic analysis and sequence divergence were used to determine the relationship of the newly discovered taxon to other *Scleroderma* species. In the phylogenetic analysis, *Scleroderma* species from broader geographic regions were included for comparison. BioEdit Sequence Alignment Editor version 7.0.9.0 was used to verify the ITS, LSU, *rpb*2, and *tef*1-*α* sequences, and SeqMan (DNAstar, Madison, WI, USA) was used to assemble the sequences. The database of the National Center for Biotechnology Information (NCBI) (http://www.ncbi.nlm.nih.gov/genbank/) was searched against each sequence using the Basic Local Alignment Search Tool (BLAST) to ensure that it belonged to the correct genus and was not contaminated and to identify the closest matches.

A GenBank BLAST search was performed to check for similarity between the newly generated sequences. In total, 82 sequences of several *Scleroderma* species from various regions, including our collections, were obtained (Table 1). *Pisolithus aurantioscabrosus*, a close relative of *Scleroderma* (Martin et al. 2002; Wilson et al. 2012), was chosen as an outgroup. Alignments were performed using MAFFT v. 7.11 (https://mafft.cbrc.jp/alignment/software/, accessed on 23 December 2023), and all alignments were trimmed separately using TrimAl to eliminate ambiguously aligned positions (Capella-Gutiérrez et al. 2009).

**Table 1.** Phylogenetic analysis list of species, herbarium number, place of origin, and GenBank accession number.

Species	Voucher information	Location	GenBank accession no.				Reference
			ITS	LSU	Rpb2	Tef1-α	
*Pisolithus aurantioscabrosus*	AWW297	Malaysia	EU718112	EU718146	FJ536648	FJ536681	Wilson et al. (2011)
*Scleroderma areolatum*	AWW211	USA	EU718115	EU718149	FJ536651	FJ536683	Wilson et al. (2011)
*S. areolatum*	PBM2208	Australia	N/A	EU718150	FJ536652	FJ536684	Wilson et al. (2011)
*S. areolatum*	TNS:F-82295	Japan	OQ025272	OQ025269	N/A	N/A	Kasuya et al. (2023)
*S. areolatum*	Kasuya-B4422	Japan	OQ025273	OQ025270	N/A	N/A	Kasuya et al. (2023)
*S. areolatum*	O3C_4	USA	JX030282	N/A	N/A	N/A	Bzdyk et al. (2018)
*S. areolatum*	23	Spain	MN684210	N/A	N/A	N/A	-
*S. areolatum*	Db-K	-	MH040288	N/A	N/A	N/A	Bzdyk et al. (2018)
*S. areolatum*	Bk-N	-	MH040301	N/A	N/A	N/A	Bzdyk et al. (2018)
*S. bermudense*	BZ3961	Belize	EU718118	DQ644137	FJ536654	FJ536686	Wilson et al. (2011)
*S. bermudense*	EUA09	-	OQ351725	N/A	N/A	N/A	Bullaín-Galardis et al. (2024)
*S. bermudense*	SUA03	-	OQ351729	N/A	N/A	N/A	Wilson et al. (2011)
*S. bovista*	MCA242	USA	EU718117	DQ644138	FJ536653	FJ536685	Wilson et al. (2011)
*S. citrinum*	AWW212	USA	EU718119	EU718151	FJ536655	FJ536687	Wilson et al. (2011)
*S. citrinum*	F-PRL5772	USA	GQ166907	N/A	N/A	N/A	Zhang et al. (2013)
*S. citrinum*	K (M) 17485	England	EU784413	N/A	N/A	N/A	Zhang et al. (2013)
*S. citrinum*	CITSCL1	USA	FM213344	N/A	N/A	N/A	Zhang et al. (2013)
*S. citrinum*	K (M) 53906	England	EU784414	N/A	N/A	N/A	Zhang et al. (2013)
*S. columnare*	CUB:Microbiology KHS3	Thailand	AB459512	N/A	N/A	N/A	Ruankaew Disyatat et al. (2016)
*S. columnare*	Scl1	Thailand	AB854700	N/A	N/A	N/A	Kaewgrajang et al. (2023)
*S. columnare*	CUB:Microbiology KHS10	Thailand	AB459519	N/A	N/A	N/A	Ruankaew Disyatat et al. (2016)
***S. columnare***	**MFLU25-0110 (DG150)**	**Thailand**	**N/A**	**N/A**	**PX137624**	**PX126632**	**This study**
***S. columnare***	**MFLU25-0111 (DG153)**	**Thailand**	**PV444716**	**N/A**	**PX137625**	**PX126633**	**This study**
*S. dictyosporum*	IR250	Burkina Faso	FJ840444	N/A	N/A	N/A	Sanon et al. (2009)
*S. dictyosporum*	IR408	Burkina Faso	FJ840445	N/A	N/A	N/A	Sanon et al. (2009)
*S. meridionale*	AWW218	USA	EU718121	EU718152	FJ536656	FJ536688	Wilson et al. (2011)
*S. mcalpinei*	OSC 24605	-	EU718122	DQ682999	FJ536657	N/A	Wilson et al. (2011)
*S. nitidum*	UFRN:Fungos 2034	Brazil	KU759904	KU759903	N/A	N/A	Raut et al. (2020)
*S. nitidum*	UFRN:Fungos 2219	Brazil	KU759908	N/A	N/A	N/A	Raut et al. (2020)
*S. polyrhizum*	AWW216	USA	EU718123	EU718153	FJ536658	FJ536689	Wilson et al. (2011)
*S. polyrhizum*	MA:fungi-39352	Spain	MT270662	N/A	N/A	N/A	Ortiz-Rivero et al. (2021)
*S. separatum*	Ge5394	China	OQ554975	N/A	N/A	N/A	Wu et al. (2023)
*S. separatum*	ZLR31	China	OQ554974	N/A	N/A	N/A	Wu et al. (2023)
*S. separatum*	Ge4148	China	OQ554973	N/A	N/A	N/A	Wu et al. (2023)
***S. separatum***	**MFLU 19-1347 (NTF066)**	**Thailand**	**PV444715**	**PV446742**	**N/A**	**N/A**	**This study**
*S. sinnamariense*	SINSCL3 (SCLN)	Thailand	FM213358	N/A	N/A	N/A	Phosri et al. (2009)
*S. sinnamariense*	150728-29	China	MH513635	N/A	N/A	N/A	Zhang et al. (2020)
*S. sinnamariense*	SINSCL1 (SCLK4)	Thailand	FM213356	N/A	N/A	N/A	Phosri et al. (2009)
*S. sinnamariense*	SINSCL6 (SCLD1)	Thailand	FM213361	N/A	N/A	N/A	Phosri et al. (2009)
*S. sinnamariense*	SINSCL4 (SCLY5)	Thailand	FM213359	N/A	N/A	N/A	Phosri et al. (2009)
*S. sinnamariense*	CMU53:210-2	Thailand	HQ687222	N/A	N/A	N/A	Kumla et al. (2014)
*S. sinnamariense*	rpr-355	-	MW374160	N/A	N/A	N/A	Wang et al. (2022)
*S. sinnamariense*	HKAS122471	China	ON794312	N/A	N/A	N/A	Wang et al. (2022)
*S. sinnamariense*	SINSCL5 (SC1)	Thailand	FM213360	N/A	N/A	N/A	Phosri et al. (2009)
*S. sinnamariense*	DX2021-8-2	-	OL351633	N/A	N/A	N/A	-
***S. sinnamariense***	**MFLU25-0112 (DG157)**	**Thailand**	**N/A**	**N/A**	**PX137633**	**PX207694**	**This study**
***S. sinnamariense***	**MFLU25-0113 (DG158)**	**Thailand**	**PV444717**	**N/A**	**PX137632**	**PX207695**	**This study**
***S. sinnamariense***	**MFLU25-0114 (DG159)**	**Thailand**	**PV444718**	**N/A**	**PX137631**	**PX137634**	**This study**
***S. sinnamariense***	**MFLU25-0115 (DG160)**	**Thailand**	**PV444719**	**N/A**	**PX137630**	**PX126634**	**This study**
***S. sinnamariense***	**MFLU 19-1647 (MO-DG020)**	**Thailand**	**PV444720**	**N/A**	**PX137626**	**PX126635**	**This study**
***S. sinnamariense***	**MFLU 19-1648 (MO-DG021)**	**Thailand**	**PV444721**	**N/A**	**N/A**	**N/A**	**This study**
***S. sinnamariense***	**MFLU 19-1649 (MO-DG022)**	**Thailand**	**PV444722**	**PV446743**	**PX137627**	**PX126636**	**This study**
***S. sinnamariense***	**MFLU 19-1650 (MO-DG023)**	**Thailand**	**PV444723**	**N/A**	**PX137628**	**N/A**	**This study**
***S. sinnamariense***	**MFLU 19-1652 (MO-DG034)**	**Thailand**	**PV444724**	**N/A**	**N/A**	**N/A**	**This study**
***S. sinnamariense***	**MFLU 19-1653 (MO-DG035)**	**Thailand**	**PV444725**	**N/A**	**PX137629**	**N/A**	**This study**
***S. sinnamariense***	**MFLU 19-1341 (NTF012)**	**Thailand**	**PV444726**	**N/A**	**N/A**	**N/A**	**This study**
*Scleroderma* sp.	AWW260	Malaysia	EU718124	EU718155	FJ536660	FJ536691	Wilson et al. (2011)
*Scleroderma* sp.	AB96	Cameroon	KR819100	N/A	N/A	N/A	Michaëlla Ebenye et al. (2017)
*Scleroderma* sp.	YAAS-L5455	-	MT876542	N/A	N/A	N/A	-
*Scleroderma* sp.	YAAS-L5449	-	MT876541	N/A	N/A	N/A	-
*Scleroderma* sp.	SL2085	Singapore	OR354966	N/A	N/A	N/A	-
*Scleroderma* sp.	ECM26-SERS	-	DQ146385	N/A	N/A	N/A	Yuwa-Amornpitak et al. (2006)
*Scleroderma* sp.	LH35	Malaysia	GQ268582	N/A	N/A	N/A	Peay et al. (2010)
***Scleroderma* sp.**	**MFLU 19-1348 (NTF090)**	**Thailand**	**PV444728**	**N/A**	**N/A**	**N/A**	**This study**
***Scleroderma* sp.**	**MFLU 19-1517 (DMSL-DG005)**	**Thailand**	**PV444729**	**N/A**	**N/A**	**PV749898**	**This study**
*S. suthepense*	AWW311	Malaysia	EU718125	EU718156	FJ536661	FJ536692	Wilson et al. (2011)
*S. suthepense*	CMU:55-SC2	Thailand	NR_132871	N/A	N/A	N/A	Kumla et al. (2013)
*S. suthepense*	JH-2016-0727-052	China	MH513626	N/A	N/A	N/A	Zhang et al. (2020)
*S. suthepense*	180508-08	China	MH513625	N/A	N/A	N/A	Zhang et al. (2020)
***S. suthepense***	**MFLU25-0109 (DG146)**	**Thailand**	**N/A**	**N/A**	**N/A**	**PX126630**	**This study**
***S. suthepense***	**MFLU 19-1344 (NTF053)**	**Thailand**	**PV444727**	**N/A**	**N/A**	**N/A**	**This study**
*S. xanthochroum*	AWW254	Malaysia	EU718126	EU718154	N/A	N/A	Wilson et al. (2011)
*S. yunnanense*	HKAS80386	-	MW493647	MW493703	N/A	N/A	Kasuya et al. (2023)
*S. yunnanense*	PERTH-7604645	China	MT270651	N/A	N/A	N/A	Ortiz-Rivero et al. (2021)
*S. yunnanense*	TNS:F-82294	Japan	OQ025271	OQ025268	N/A	N/A	Kasuya et al. (2023)
uncultured fungus	ASV_419	-	LR993736	N/A	N/A	N/A	-
uncultured fungus	ASV_1014	-	LR994331	N/A	N/A	N/A	-
***Scleroderma longistipes***	**MFLU 19-1655 (DG109)**	**Thailand**	**PV444712**	**PV446740**	**PX126607**	**PX121227**	**This study**
***Scleroderma longistipes***	**MFLU 19-1656 (DG110)**	**Thailand**	**PV444713**	**PV446741**	**PX121228**	**N/A**	**This study**
***Scleroderma magnisporum***	**MFLU 19-1345 (NTF062)**	**Thailand**	**PV444714**	**N/A**	**N/A**	**N/A**	**This study**
***Scleroderma microcarpum***	**MFLU 19-1349 (DG002)**	**Thailand**	**PV436898**	**N/A**	**N/A**	**N/A**	**This study**

N/A: not available; species in this study are indicated in bold black.

The character sets included 78 collections and 676 characters (including gaps) from ITS, 20 collections and 1402 characters from LSU, 20 collections and 1120 characters from *rpb*2, and 20 collections and 1035 characters from *tef*1-*α*. The final dataset comprised 82 collections and 4233 characters from ITS+LSU+*rpb*2+*tef*1-*α*. After checking for unsupported conflicts (BS < 70%) between single-gene maximum likelihood (ML) phylogenies, a concatenated four-locus dataset was assembled.

Phylogenetic analysis using ML was performed, followed by manual adjustments in raxmlGUI 2.0.13, along with Bayesian analysis, both conducted on the CIPRES Science Gateway version 3.3 web server (Miller et al. 2010), available at https://www.phylo.org/. A mixed-model (partitioned) scheme was employed for both ML and Bayesian analyses, with the alignment split into four-character sets: ITS, LSU, *rpb2*, and *tef*1-*α*. The best-fit substitution models of jModelTest2 version 2.1.6 (Darriba et al. 2012) in XSEDE were chosen for Bayesian analysis. The selected models were HKY+G for ITS, GTR+I+G for nrLSU, HKY+G for *rpb*2, and SYM+I+G for *tef*1-*α*. Four independent runs, each with four chains, were performed for 1,000,000 generations, with sampling every 100 generations. The average standard deviation of split frequencies at the end of the runs was 0.015009. The burn-in phase (25%) was determined by assessing stationarity in the generation-likelihood plot using Tracer version 1.7.1 (Rambaut et al. 2018). The resulting phylogenetic tree was visualized in Treeview 32 and further edited using Adobe Illustrator CS6.0.

## ﻿Results and discussion

Based on *Scleroderma* species, they were formerly separated by morphology, with basidiome size and shape varying depending on soil and environment and basidiospore morphology (Watling 2006; Sanon et al. 2009; Kumla et al. 2013; Gonkhom et al. 2025). In this study, *Scleroderma* sp. nov., described from macromorphological and micromorphological traits together with phylogenetic analysis of ITS, LSU, *rpb*2, and *tef*1-*α* genes, includes three new species—*Scleroderma longistipes*, *Scleroderma magnisporum*, and *Scleroderma microcarpum*—from northern Thailand. This work enhances our understanding of the diversity of *Scleroderma* species. Macroscopically, the size, color, and type of dehiscence of the basidiome, as well as the color and thickness of the peridium, are crucial traits for identifying *Scleroderma*. Microscopically, the size, shape, and ornamentation of the basidiospores are employed to differentiate species of this genus. The basidiomes and basidiospores are similar in size and character to those of *S. separatum*, *S. dictyosporum*, and *S. hypogaeum* (Sims et al. 1995; Cortez et al. 2011; Guzmán et al. 2013). Our ITS, LSU, *rpb*2, and *tef*1-*α* sequence analyses clearly separate these species from other reticulate-spored *Scleroderma* species in the section.

### ﻿Phylogenetic analysis

**Figure 1.**
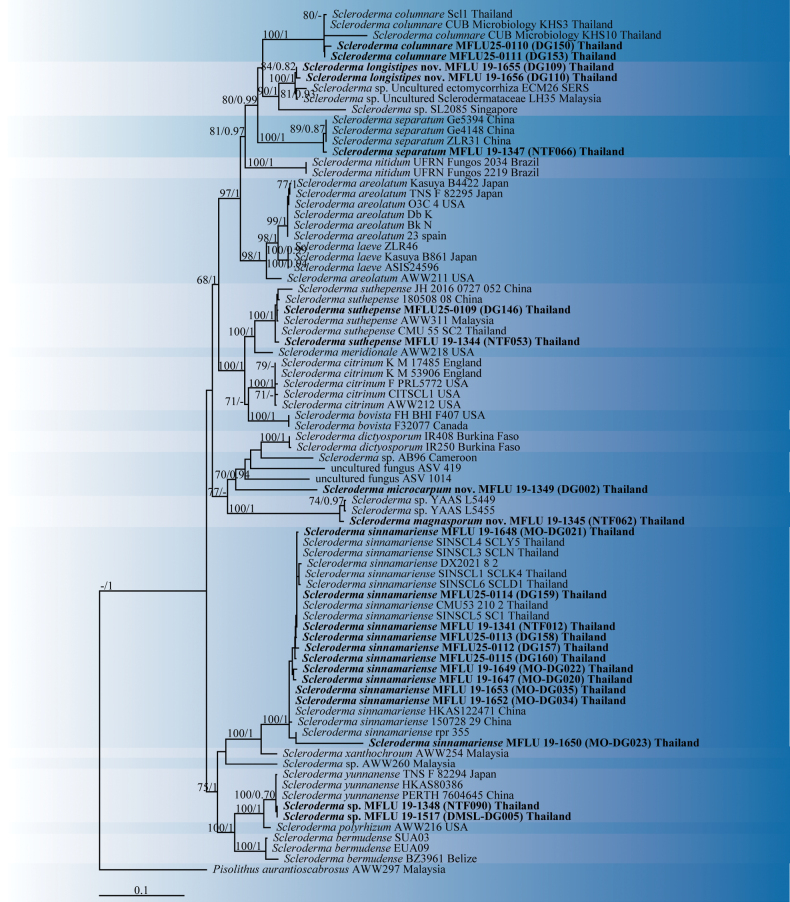
Phylogenetic tree obtained from the maximum likelihood analysis of *Scleroderma* species. Maximum likelihood tree obtained from the alignment of ITS, LSU, *rpb*2, and *tef*1-α sequences. The bootstrap consensus tree was inferred from 1000 replicates. *Pisolithus aurantioscabrosus* was included as an outgroup.

The combined dataset of four genes comprised 4233 bp (including gaps): 676 bp for ITS, 1402 bp for LSU, 1120 bp for *rpb*2, and 1035 bp for *tef*1-*α*. The best RAxML phylogram, with a final likelihood value of -22414.937168, is presented. The matrix had 1563 distinct alignment patterns with 68.28% undetermined characters or gaps. The estimated base frequencies were: A = 0.233328, C = 0.255317, G = 0.274489, and T = 0.236866. Substitution rates were AC = 1.330307, AG = 4.855565, AT = 1.388503, CG = 1.501262, CT = 8.964382, and GT = 1.000000. The gamma distribution shape parameter was α = 0.929050. The phylogram topology derived from Bayesian analysis was similar to that from ML analysis. Bootstrap values of ML ≥ 70% and Bayesian posterior probabilities (PP) ≥ 0.90 are indicated in Fig. 1.

Phylogenetic trees inferred from ML and MrBayes analyses resulted in similar topologies; therefore, only the ML tree (ITS+LSU+*rpb*2+*tef*1-*α*) is shown, with both ML bootstrap (BS) values and Bayesian posterior probabilities (PP). In the phylogram, *Scleroderma longistipes* (MFLU 19-1655 and MFLU 19-1655) was closely related to *Scleroderma* sp. (uncultured ectomycorrhiza ECM26 SERS, LH35 from Malaysia, and SL2085 from Singapore) with high statistical support: 100% BS/1.00 PP, 81% BS/0.93 PP, and 90% BS/1.00 PP, respectively. *Scleroderma microcarpum* (MFLU 19-1347) was closely related to the uncultured fungi ASV 1014 and ASV 419, with statistical support of 70% BS/0.94 PP. *Scleroderma magnisporum* (MFLU 19-1345) was closely related to *Scleroderma* sp. YAAS L5449 and YAAS L5455, with statistical support of 74% BS/0.97 PP.

The species most closely related in the phylogenetic tree to *Scleroderma microcarpum* (MFLU 19-1347) was *S. dictyosporum* (Voucher IR250), with a genetic distance of 11.81% (65/570) between ITS sequences. *Scleroderma magnisporum* (MFLU 19-1345) and *S. microcarpum* (MFLU 19-1347) showed a genetic distance of 21.34% (95/445), supporting their distinction as separate species. This result is consistent with previous molecular phylogenetic studies that strongly support the recognition of *Scleroderma* species as genetically discrete lineages (Phosri et al. 2009; Nouhra et al. 2012; Wu et al. 2023).

*Scleroderma* species are found in temperate, tropical, and subtropical regions, which may be related to the higher diversity of *Scleroderma* or ectomycorrhizal fungi in these climatic zones (Jeffries 1999; Brundrett et al. 2005; Pradhan et al. 2011; Wu et al. 2023). In addition, 11 species of *Scleroderma* (*S. areolatum*, *S. bovista*, *S. cepa*, *S. citrinum*, *S. dictyosporum*, *S. flavidum*, *S. lycoperdoides*, *S. polyrhizum*, *S. sinnamariense*, *S. verrucosum*, and *S. suthepense*) have been recorded in Thailand based on morphology (Chandrasrikul et al. 2011; Kumla et al. 2013; Gonkhom et al. 2025). Phylogenetic analysis confirmed the placement of *Scleroderma separatum* based on the ITS and LSU regions (Fig. 1). *S. separatum* exhibits similar shapes and sizes of basidiomes and basidiospores (Wu et al. 2023). Phylogenetic analysis based on ITS and LSU sequences facilitated confirmation of the species we analyzed, which has been officially recorded in Thailand.

### ﻿Taxonomy

#### ﻿ Scleroderma longistipes

Taxon classificationFungiBoletalesSclerodermataceae

Gonkhom, Sysouph. & Thongkl. sp. nov.

1984DD66-41C7-5225-80E5-08BC5BA01C9C

Index Fungorum: IF903880

Fig. 2

##### Diagnosis.

Epigeous brown to burnt umber basidiomata with long stipe, rubbery pale brown peridium, hyaline to yellow brown hyphae in exoperidium, hyaline hyphae in endoperidium, globose dark brown basidiospores with echinulate or spinose ornamentation.

##### Holotype.

Thailand • Chiang Rai Province, Mueang Chiang Rai District, Mae Fah Luang University campus, 04 June 2019, collected by Didsanutda Gonkhom, DG109 (MFLU 19-1655).

##### Etymology.

The species name (*longistipes*) refers to the long stipe of the basidiomata.

##### Description.

Basidiomata epigeous, 28–35 mm in diam., 42–60 mm high, club-shaped, with globular peridial head; with cracked to squamulose surface, brown (6E5) background when young, with fawn (7E4) to brown (6E5) or burnt umber (6F6) squamular cracks upon luteous background, hard skin, tough when mature. Stipe sub-cylindric, fat, with small irregular cracks at the top of the strip, 35–45 × 10–13 mm, white background, covered with brown (6E5) fibrillose squamules. Rhizomorphs more aggregated at the base, white, branched, and narrowing towards the base. Context white in peridium and stipe, turned dull red to greyish red (98B4-5) when cut. Peridium up to 5 mm wide when fresh, rubbery in consistence, pale brown (6D5).

Peridium layer formed by simple-septate hyphae. Exoperdium slightly thickened walls, composed of interwoven to ramified and superimposed hyphae, hyaline to yellow brown, 2.9–3.8 µm diam. Endoperdium thick, composed of interwoven hyphae, hyaline, 4.3–7.2 µm diam. Clamp connections present on endoperidium hyphae. Gleba brownish grey (9E2), greyish brown (9E3), or oxblood red (9E7) to dark brown (9F4-7), compact, and powdery when mature. Basidiospores (n = 50) globose, echinulate, dark brown in KOH, (13.4–)14.5–17.2(19.5) µm in diam., with brown spinose ornamentation (2.6–5.9 µm high). Basidia not seen.

**Figure 2.**
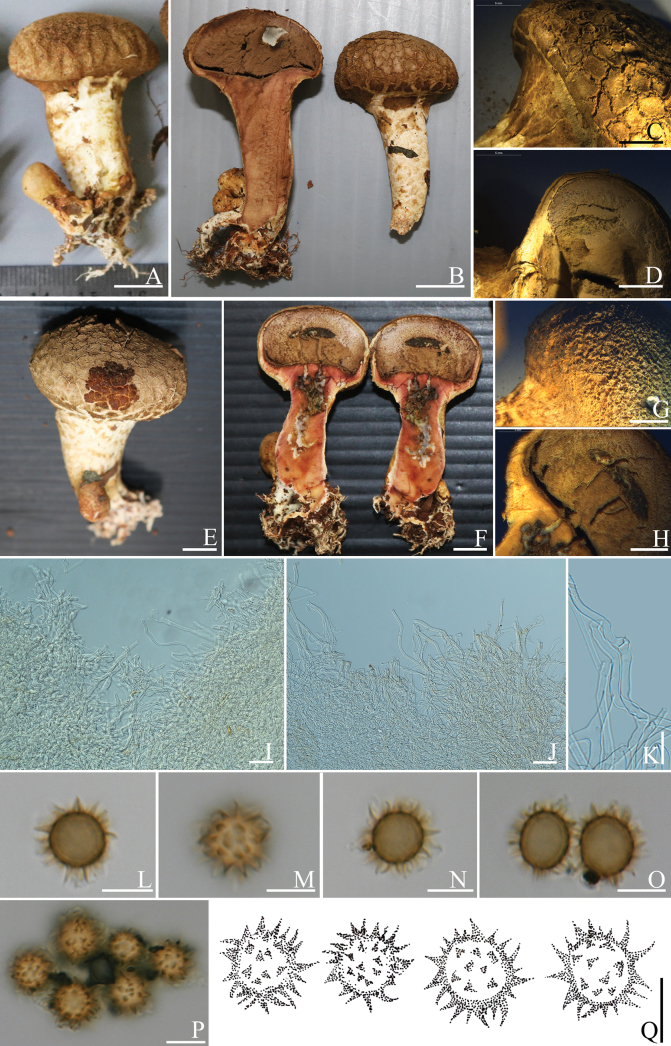
*Scleroderma longistipes* (MFLU 19-1655, holotype). A, B. Basidiomata; C. Scale on peridium surface; D. Cut side of peridium of MFLU 19-1655; E, F. Basidiomata; G. Scale on peridium surface; H. Cut side of peridium of *Scleroderma longistipes* (MFLU 19-1656); I. Exoperidial hyphae; J. Endoperidial hyphae; K. Clamped hyphae of endoperidium; L–Q. Basidiospore. Scale bars: 10 mm (A, B, E, F); 5 mm (C, D, G, H); 50 µm (I, J); 20 µm (K); 10 µm (L–Q).

##### Habitat and distribution.

Caespitose or fasciculated on soil, epigeous, in northern Thailand.

##### Additional specimens examined.

Thailand • Chiang Rai Province, Mueang Chiang Rai District, Mae Fah Luang University campus, 04 June 2019, collected by Didsanutda Gonkhom, DG110 (MFLU 19-1656).

##### Notes.

*Scleroderma longistipes* is characterized by a larger brown basidiomata with a longer stipe that turns from dull red to greyish red when touched. The basidiospores are globose with longer brown spines. According to the phylogenetic analysis (Fig. 1), Thai specimens of *Scleroderma longistipes* are identical to an unknown species from Malaysia (LH35) (Peay et al. 2010) and an unknown species from Thailand (ECM26-SERS) (Yuwa-Amornpitak et al. 2006). However, these two taxa were only identified as Sclerodermataceae species. *Scleroderma separatum* Z.W. Ge, R. Wu & L.R. Zhou, a species originally described from Yunnan, southwestern China, is a species related to *S. longistipes* by having a stipe. However, *Scleroderma longistipes* appears closely related to *S. separatum*, has smaller basidiomata, a greenish yellow background, a slender stipe (5–30 × 3–5 mm), smaller basidiospores (4.5–8.5 µm), and shorter basidiospore spines (1.2–2.5 µm) (Wu et al. 2023). Furthermore, *S. separatum* is related to *S. longistipes* by phylogenetic analysis with low bootstrap support (BS) (Fig. 1).

*Scleroderma longistipes* is also similar to *S. columnare* Berk. & Broome. However, *S. columnare* has stellate dehiscence at the upper part of basidiomata in old specimens (London 1911), and *S. columnare* is also related to *S. longistipes* with low BS (Fig. 1). Additionally, *S. nitidum* Berk. is morphologically similar to *S. longistipes*, sharing the stipitate morphology but differing in having a glossy peridium, smaller basidiospores (5–7 µm) with denser, shorter spines, and no color change when bruised (Guzmán 1970).

#### ﻿ Scleroderma microcarpum

Taxon classificationFungiBoletalesSclerodermataceae

Gonkhom, Sysouph. & Thongkl. sp. nov.

15A82431-29F2-5AF5-880D-C8A2795B5243

Index Fungorum: IF903881

Fig. 3

##### Diagnosis.

Different from the similar species *S. dictyosporum* in having smaller basidiomata and larger basidiospores.

##### Holotype.

Thailand • Chiang Mai Province, Mae On District, 9 October 2019, collected by Didsanutda Gonkhom, DG002 (MFLU 19-1347).

##### Etymology.

The species name “*microcarpum*” refers to the small size of the basidiomata.

##### Description.

Basidiomata epigeous, 20–21 mm in diam. 10–20 mm in height, circular when young, depressed at maturity; surface smooth and with small scales when young stage, light yellow (5A4-5), covered with brown (7E6-8) squamules when mature. Stipe sessile or short pseudostipitate (less than 4 mm long). Rhizomorphs at the base, white, branched, narrowing towards the base. Context up to 2 mm thick, light yellow (5A4-5).

**Figure 3.**
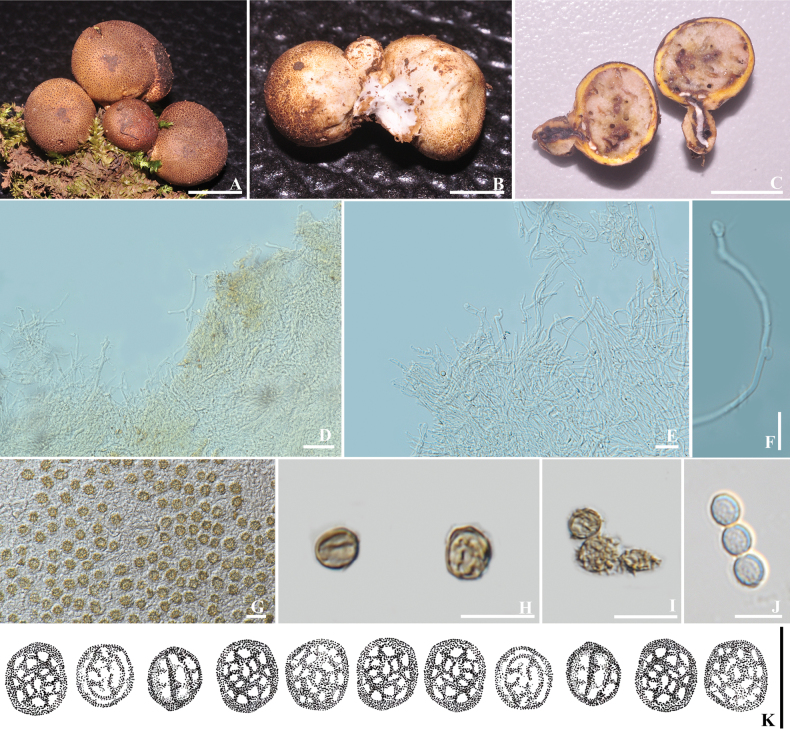
*Scleroderma microcarpum* (MFLU 19-1347, holotype). A, B. Basidiomata; C. Context of peridium; D. Exoperidial hyphae; E. Endoperidial hyphae; F. Clamped hyphae of endoperidium; G–K. Basidiospore. Scale bars: 10 mm (A–C); 20 µm (D); 50 µm (E); 20 µm (F); 10 µm (G–K).

Peridium layer formed by simple hyphae septate, hyaline to yellow brown. Exoperdium 6.9–9.3 µm in diam., with clamp connections. Endoperidium 4.2–8.2 µm in diam., with or without clamp connections. Gleba white – yellowish white (3A1-2), compact, and powdery when mature. Basidiospores (n = 50) globose to subglobose, echinulate, grayish brown in KOH, (3.97–)6.07–6.51(–8.15) × (8.24–)10.77–11.61(–13.33) µm in diam. Basidia not observed.

##### Habitat and distribution.

Caespitose or fasciculated on soil, epigeous, in northern Thailand.

##### Known distribution.

Northern Thailand.

##### Note.

*Scleroderma microcarpum* is characterized by small basidiomata with a smooth and small scale on the surface and larger basidiospores and globose echinulate. *Scleroderma microcarpum* is phylogenetically related to *S. dictyosporum* Pat. with low BS (Fig. 1). Both species have echinulate basidiospores. However, *S. dictyosporum* has a larger basidiomata (24–28 mm in diam.) (Sanon et al. 2009) and has smaller basidiospores (7–9 µm wide) (Patouillard 1896; Sanon et al. 2009).

#### ﻿ Scleroderma magnisporum

Taxon classificationFungiBoletalesSclerodermataceae

Gonkhom, Sysouph. & Thongkl. sp. nov.

037560B7-F429-56FC-B22A-359D821FC5BC

Index Fungorum: IF903882

Fig. 4

##### Diagnosis.

Epigeous basidiomata with irregular club shape, smooth to slightly cracked to squamulose peridial head, brown to burnt umber, sessile or short pseudostipitate stipe, white, pale brown context, with hyphae simple-septate in both endoperidium and exoperidium, dark brown globose to subglobose basidiospores with crowded spines.

##### Holotype.

Thailand • Chiang Rai Province, Mueang Chiang Rai District, 16 July 2010, collected by Naritsada Thongklang, NTF062 (MFLU 19-1345).

##### Etymology.

The species name “*magnisporum*” refers to its larger basidiospores.

##### Description.

Basidiomata epigeous, 35 mm in height, 22–34 mm in diam., club-shaped, with an irregularly globular peridial head; surface smooth, slightly cracked to squamulose, brown (6E5) to burnt umber (6F6), on pale orange (5A3) background. Sessile or short pseudostipitate (10 mm high), brown (6E5). Context thick, up to 5 mm wide. Exoperidium composed of hyphae simple-septate, interwoven, hyaline to yellow, 4.1–5.8 µm in diam. Endoperidium layer formed by hyphae simple-septate, with slightly thickened walls, interwoven, hyaline, 5.8–9.6 µm in diam., and hyphae from the endoperidium toward the gleba are pale yellow (4A3) and black in the mature gleba. Clamp connections present on endoperidium hyphae. Basidiospores (n = 50) globose to subglobose, echinulate with crowded curved spines, dark brown in KOH, (7.67–)12.42–8.42(–13.46) × (10.33–)14.53–11.42(–15.50) µm including ornamentation. Basidia not seen.

**Figure 4.**
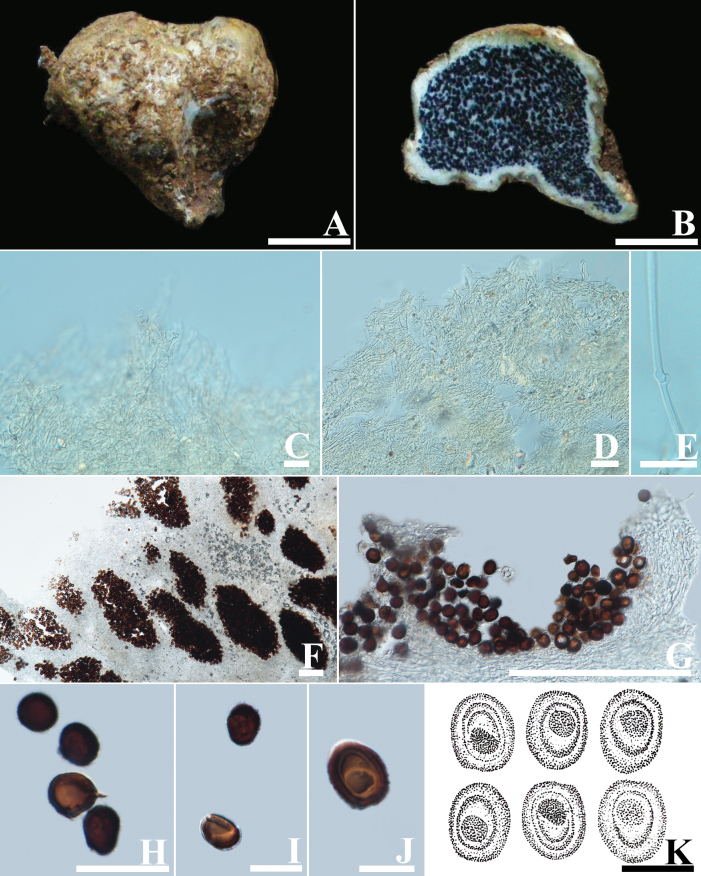
*Scleroderma magnisporum* (MFLU 19-1345, holotype). A. Basidiomata; B. Cut side of the peridium; C. Exoperidial hyphae; D. Endoperidial hyphae; E. Clamped hyphae of endoperidium; F–K. Basidiospore. Scale bars: 10 mm (A, B); 20 µm (C–E); 50 µm (F–G); 20 µm (H); 10 µm (I–K).

##### Habitat and distribution.

Solitary on soil, epigeous, in northern Thailand.

##### Known distribution.

Northern Thailand.

##### Note.

*Scleroderma magnisporum* is characterized by a smooth, slightly cracked surface and larger basidiospores. The microcharacter of *S. magnisporum* is similar to that of *S. hypogaeum* Zeller. However, *S. hypogaeum*, originally described from Oregon, has a smooth, slightly cracked, or subscaly basidiome, with larger basidiospores up to 22–30 μm diam. (Zeller 1922; Guzmán et al. 2013). *S. magnisporum* is phylogenetically close to *Scleroderma microcarpum* (MFLU 19-1347) in this study (Fig. 1). Both species are clearly different in their basidiomata size and shapes; the basidiomata of *S. microcarpum* are much smaller than those of *S. magnisporum* (7.6–15.5 µm diam.). While *S. yunnanense* shares with *S. magnisporum* a smooth to faintly cracked peridium and large basidiospores (15–20 μm), it has a pale yellow to ochre peridium (Guzmán 1970; Zhang et al. 2013).

#### ﻿ Scleroderma separatum

Taxon classificationFungiBoletalesSclerodermataceae

Z.W. Ge, R. Wu & L.R. Zhou.

6C8A789B-8308-5BB3-99A0-16E65B394F50

Index Fungorum: IF847687

Fig. 5

##### Description.

Basidiomata are epigeous, 12–28 mm in diam., 17–45 mm in height, globose, subglobose to irregularly oblate, tan (3D3) to ochraceous–brown (5E2). Peridium is leathery, thin, 0.5–1.0 mm thick when fresh, and becomes much thinner when dry, hay (5C4) to greenish-yellow (3B4) background. Peridium layer formed by hyphae simple-septate, with slightly thickened walls, interwoven, hyaline, exoperidium 2.4–4.4 µm in diam., and endoperidium 4.1–6.0 µm in diam. with clamp connections. Gleba grey (8F1) –dark brown (8F5), compact, and powdery when mature. Stipe is subcylindric, 20–50 mm in length and 5–10 mm in diam., with numerous white rhizomorphs at the base. Basidiospores globose, occasionally subglobose, dark brown in KOH, (12.31–)13.23–14.35 (–16.49) × (11.53–)13.78–14.29(–16.30) µm in diam., including ornamentation (spinose up to 1.7–3.9 µm high), n = 50, coated by crowded curved spines. Basidia not seen.

**Figure 5.**
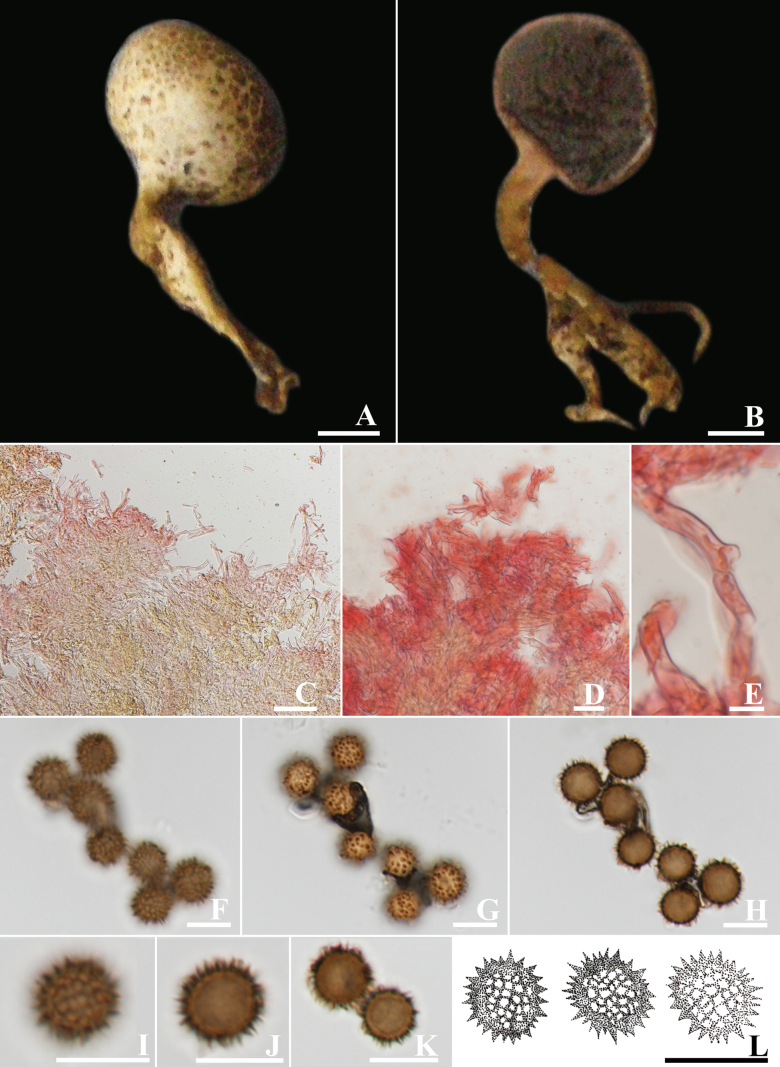
*Scleroderma separatum* (MFLU 19-1347). A. Basidiomata; B. Cut side of the peridium; C. Exoperidial hyphae; D. Endoperidial hyphae; E. Clamped hyphae of endoperidium; F–L. Basidiospore. Scale bars: 10 mm (A, B); 50 µm (C); 20 µm (D); 10 µm (E); 20 µm (F–L).

##### Habitat and distribution.

Caespitose or fasciculated on soil, epigeous, in tropical and temperate regions of China and Thailand.

##### Specimens examined.

Thailand • Chiang Mai Province, Mae Rim District, Mae Sa, 28 July 2010, MFLU 19-1347 (NTF066).

##### Note.

This is based on a single Thai specimen. Thai specimens are considered to be similar to specimens of *S. separatum* from Southwestern China by having epigeous basidiomata. The molecular analysis also supports identification (Fig. 1). The species grows under a forest dominated by *Pinus yunnanensis* (Wu et al. 2023). Nonetheless, the samples from Thailand were frequently taken from mixed forests or the litter of *Pinus* trees. These results suggest that this species is found on both *Quercus* and *Pinus*. Since the initial description, this is the second record of the species.

**Figure 6.**
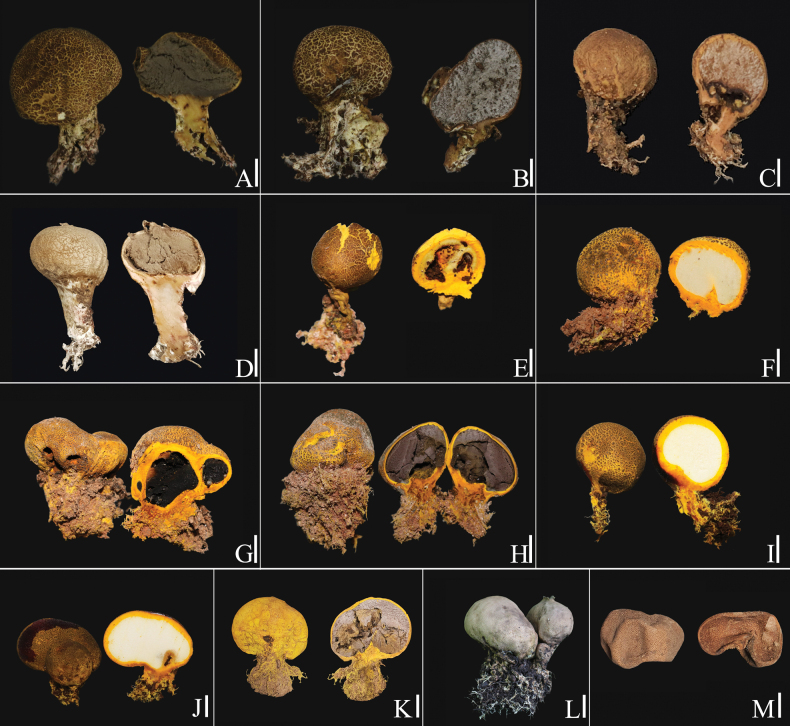
The mature basidiomata of *Scleroderma* spp. in this study. A, B. *S. columnare*; C–K. *S. sinnamariense*; L, M. *S. suthepense.* Scale bars: 10 mm.

Among 82 accessions, including the newly described species, the length of the entire ITS, LSU, *rpb*2, and *tef*1-*α* comprised 4233 base pairs. The *Scleroderma* species known from this study include *Scleroderma columnare*, *S. sinnamariense*, and *S. suthepense* (Fig. 6).

### ﻿Key to *Scleroderma* species in Thailand

**Table:** 

1	Basidiome sessile or with a short pseudostipe	**2**
–	Basidiome with a well-developed pseudostipe or stalk-like base	**10**
2	Basidiome globose to subglobose, peridium thick (1–2 mm)	**3**
–	Basidiome irregularly shaped, peridium thin (<1 mm)	**5**
3	Peridium yellowish to orangish-yellow, smooth to cracked	***S. cepa***
–	Peridium brown, with distinct warts or scales	**4**
4	Peridium covered with tough raised warts, yellow-brown	***S. citrinum***
–	Peridium cracked, scaly, roughened, brown	***S. bovista***
5	Peridium thin, leathery, yellowish white	**6**
–	Peridium thick, with distinct cracks or subscaly	**8**
6	Basidiospores 7–8.5 µm, dark brown, reticulate	***S. dictyosporum***
–	Basidiospores larger than 8.5 µm, spiny	**7**
7	Basidiospores 8–12 µm, round, net-like ridges	***S. verrucosum***
–	Basidiospores 10–15 µm, slightly roughened texture	***S. columnare***
8	Peridium smooth, brown, tough, thick (up to 5 mm)	***S. magnisporum***
–	Peridium cracked or scaly, background yellowish	**9**
9	Basidiospores 8.24–13.33 µm, rhizomorphs pale brown	***S. microcarpum***
–	Basidiospores 12.31–16.49 µm, spines up to 3.9 µm	***S. separatum***
10	Stipe well-developed, more than 3 cm long	**11**
–	Stipe short, less than 3 cm, or absent	**13**
11	Stipe sub-cylindric, cracked at the top, basidiospores 13.4–19.5 µm	***S. longistipes***
–	Stipe short or irregular, basidiospores smaller (<14 µm)	**12**
12	Basidiospores 7–12 µm, peridium star-shaped when split	***S. polyrhizum***
–	Basidiospores 8–13 µm, peridium smooth to scaly	***S. suthepense***
13	Peridium golden yellow, apex rupturing at maturity	***S. flavidum***
–	Peridium brown to ochraceous, scaly, or roughened	**14**
14	Peridium leathery, verrucose, yellowish to lemon-yellow	***S. sinnamariense***
–	Peridium brownish, smooth to scaly, spore mass dark brown	**15**
15	Basidiome surface smooth, spore mass clearly olive	***S. lycoperdoides***
–	Basidiome rough or scaly, spore mass brown to dark brown	***S. areolatum***

Overall, this study significantly advances our understanding of the diversity and taxonomy of *Scleroderma* species in northern Thailand, describing three new species—*Scleroderma longistipes*, *S. microcarpum*, and *S. magnisporum*—and reporting a new record, *S. separatum*, from Thailand. The research integrates comprehensive morphological analyses with molecular phylogenetic methods, utilizing sequences from four loci (ITS, LSU, *rpb*2, and *tef*1-*α*) to confirm the distinctiveness of these taxa. The findings contribute to our knowledge of *Scleroderma* and establish a basis for future ecological and evolutionary research on ectomycorrhizal fungi.

## Supplementary Material

XML Treatment for Scleroderma longistipes

XML Treatment for Scleroderma microcarpum

XML Treatment for Scleroderma magnisporum

XML Treatment for Scleroderma separatum

## ﻿Additional information

### Use of AI

No use of AI was reported.

### Author ORCIDs

Didsanutda Gonkhom https://orcid.org/0000-0002-4827-9771

Phongeun Sysouphanthong https://orcid.org/0000-0002-1275-2459

Marc Stadler https://orcid.org/0000-0002-7284-8671

Naritsada Thongklang https://orcid.org/0000-0001-9337-5001

Kevin D. Hyde https://orcid.org/0000-0002-2191-0762

### Data availability

All of the data that support the findings of this study are available in the main text.
